# MiR-183-5p promotes migration and invasion of prostate cancer by targeting TET1

**DOI:** 10.1186/s12894-023-01286-7

**Published:** 2023-07-10

**Authors:** Yuehua Feng, Kai Wang, Minchao Qin, Qianfeng Zhuang, Zhen Chen

**Affiliations:** 1 Clinical Medical Research Center, The Third Affiliated Hospital of Soochow University, Changzhou, Jiangsu China; 2 Department of Urology, Sir Run Run Hospital, Nanjing Medical University, Nanjing, Jiangsu China; 3 Department of Urology, The Third Affiliated Hospital of Soochow University, Changzhou, Jiangsu China

**Keywords:** Prostatic cancer, miR-183-5p, Migration, Invasion

## Abstract

**Background:**

Prostate cancer (PCa) is one of the common malignant tumors worldwide. MiR-183-5p has been reported involved in the initiation of human PCa, this study aimed to investigate whether miR-183-5p affects the development of prostate cancer.

**Methods:**

In this study, we analyzed the expression of miR-183-5p in PCa patients and its correlation with clinicopathological parameters based on TCGA data portal. CCK-8, migration assay and invasion and wound-healing assay were performed to detect proliferation, migration and invasion in PCa cells.

**Results:**

We found the expression of miR-183-5p was significantly increased in PCa tissues, and high expression of miR-183 was positively associated with poor prognosis of PCa patients. Over-expression of miR-183-5p promoted the migration, invasion capacities of PCa cells, whereas knockdown of miR-183-5p showed reversed function. Furthermore, luciferase reporter assay showed TET1 was identified as a direct target of miR-183-5p, which was negatively correlation with miR-183-5p expression level. Importantly, rescue experiments demonstrated TET1 over-expression could reverse miR-183-5p mimic induced-acceleration of PCa malignant progression.

**Conclusion:**

Our results indicated that miR-183-5p could act as a tumor promoter in PCa and it accelerated the malignant progression of PCa by directly targeting and down-regulating TET1.

**Supplementary Information:**

The online version contains supplementary material available at 10.1186/s12894-023-01286-7.

## Introduction

Prostate cancer (PCa), is the second most common malignancy in men worldwide (only behind lung cancer), accounting for over 1,400,000 new cases annually, and causing around 350,000 mortalities [1, 2]. PCa often affects the older males with ages over 50 years [3]. Although PCa usually evolves slowly during the early stages, metastatic progression severely worsens patient prognosis and leads to death [4]. Hence, new therapies that target advanced disease are urgently needed.

Serum prostate-specific antigen (PSA) measurements are widely utilized in PCa diagnosis, but the specificity of PSA testing is unfortunately poor, the positive predictive value of PSA is only 24–37% [5–7]. Single use of PSA testing leads to overdiagnosis and overtreatment, so its extensive use is not recommended. Therefore, basic research has been focused on searching for new biomarkers able to provide the efficient diagnosis and to predict cancer progression.

MicroRNAs (miRNAs) are a kind of non-coding RNAs with 22–25 nucleotides encoded by endogenous genes. MiRNAs have been reported involving in the initiation and progression of PCa [8], such as miR-222 [9, 10], miR-141 [11, 12], miR-145 and miR-148 [13, 14]. MiR-183-5p is a recently discovered cancer-related miRNA, which has been confirmed to be an oncogenic role in PCa [15–18]. Studies reported that the expression of miR-183-5p in prostate biopsy was correlated with tumor status and prognosis [19, 20]. Moreover, miRNA-183 expression could also reduce tumor cell adhesion of PCa [21]. Furthermore, miR-183 positively regulated the level of prostate-specific antigen (PSA) in serum and it might be used as a prognostic marker of diagnosis progression of PCa [15, 22, 23]. Similarly, in our previous experiments, we found the expression of miR-183-5p was up-regulated in PCa tissues.

TET1 is a member of the Ten-eleven-translocation enzymes (TETs) family. It is a DNA demethylase that hydroxylates 5-methylcytosine (5mC) to 5-hydroxymetylcytosine (5hmC), thereby facilitates active DNA demethylation [8, 24]. Studies confirmed that abnormalities in DNA methylation are crucial in tumor formation, and the dysregulation of TET1 level was found in multiple malignancies such as gastric, lung, breast cancer [25–29]. Though reduction of TET1 expression in most tumors was once considered a hallmark of cancer, recent studies have shown that high TET1 expression was associated with tumor grade and poor outcome in triple-negative breast cancer (TNBC) [30]. All these results suggest that TET1 may serve as both an oncogenic role and a tumor suppressor.

In our study, we hypothesize that miR-183-5p could promote migration and invasion of PCa by regulating the target gene TET1, thereby exacerbating the process of PCa.

## Materials and methods

### PCa tissues samples

A total of 12 prostate cancer tissues and their paired adjacent tissues were collected from patients undergoing the radical prostatectomy at the Third Affiliated Hospital of SooChow University from Jun 2019 to Dec 2019. Patients enrolled in this study received no preoperative radiotherapy and/or chemotherapy. Each tissue was snap-frozen immediately in liquid nitrogen after excision, and stored at -80ºC until use.

### Bioinformatic analyses

Gene expression and clinicopathological features of patients with prostate cancer in PRAD-TCGA were downloaded from UCSC Xena Cancer browser (https://xenabrowser.net). Microarray datasets (GSE21036 and GSE64318) containing miR-183-5p expression profiles of prostate biopsy samples (52 cancer tissues and 52 adjacent tissues) were downloaded from Gene Expression Omnibus (GEO) (https://www.ncbi.nlm.nih.gov/gds/). SPSS17.0 (Chicago, IL, USA) and GraphPad Prism 5.0 (San Diego, CA, USA) software was used for the statistical analysis. Moreover, TargetScan, miRDB, miRWalk and StarBase were used to predict miR-183-5p target genes.

### Cell lines and transfection

Human PCa cell lines, including androgen-dependent LNCaP and androgen-independent PC-3, were obtained from the Cell Bank of the Committee on Type Culture Collection of the Chinese Academy of Sciences. Cells were cultured in F12K or RPMI-1640 medium (Gibco, Carlsbad, CA) containing 10% fetal bovine serum (BI, Israel) at 37ºC with 5% CO_2_.

MiR-183-5p mimics, mimics negative control (miR-NC), miR-183-5p inhibitor and inhibitor NC were purchased from GenePharma (GenePharma, Shanghai, China). Cells were seeded in 6-well plates and grown to a density of 40–60%, then transfection was performed using Lipofectamine 2000 (Invitrogen, CA, USA) according to the manufacturer’s instructions, respectively. And these cells were harvested for verification of transfection efficacy after 48 h.

### CCK-8 assay

Cells were seeded in a 96-well plate at a density of 1 × 10^4^ cells/well, and culture medium was used as a blank control. CCK-8 was added to each well (six replicate wells per group) at the indicated time points (10 µl/well), and the absorbance at 450 nm was measured 2 h later to estimate viable cells using an automatic plate reader.

### Cell migration and invasion assay

A transwell chamber with an 8-µm pore size polycarbonate membrane (Corning, NY) was used to evaluate cell migration. After 48 h of transfection, the PCa cells were resuspended with basic medium and seeded into the upper chamber (2 × 10^4^ for PC-3 cells and 5 × 10^4^ for LNCaP cells), while culture medium containing 15% FBS was added to the lower chamber. After incubation for 48 h at 37 °C, the medium was removed from the upper chamber, and non-migrated cells were scraped off with a cotton swab. The migrated cells on the other side of the membrane were fixed in 4% paraformaldehyde for 30 min, stained with crystal violet for 15 min, and counted under an inverted microscope at 200x magnification in at least three randomly selected fields.

In the invasion assay, the upper chamber was pre-coated with 10% Matrigel (BD Biosciences, San Jose, CA, United States) and the procedures of the cell invasion assay were identical to the cell migration assay.

### Wound healing assay

Transfected cells were plated into 6-well plates and grown to 90% confluence. A 10 µl sterile pipette tip was used to create a scratch wound across cell monolayer, then detached cells and debris were removed by PBS, and the attached cells were incubated in the medium with 1% FBS. Images of the wound closure was captured at 0 and 48 h using the microscope.

### Luciferase reporter assay

To verify whether miR-183-5p directly binds to the 3’UTR of TET1, luciferase reporter assay was performed. MiR-183-5p mimics or mimics negative control (NC) were transfected into HEK293T cells together with TET1 (wild type (WT) or mutant (MUT)) using lipofectamine 2000 (Invitrogen), respectively. The relative luciferase activities were measured using a detection kit (Promega Corp, USA) after 48 h, according to the manufacturer’s instructions.

### Western blot analysis

The cells were harvested and lysed using protein RIPA buffer (Beyotime, Shanghai, China) supplemented with protease inhibitor and phosphatase inhibitor (Thermo Fisher Scientific, USA). The protein concentration was determined by the BCA protein assay kit (Beyotime, Shanghai, China). Samples were mixed with SDS-PAGE loading buffer (Beyotime, Shanghai, China) and separated by 10% SDS-polyacrylamide gel electrophoresis (SDS-PAGE). After transferring onto polyvinylidene difluoride (PVDF) membranes (Merck Millipore, Billerica, MA, USA), the membranes were blocked with 5% skim milk in TBST at room temperature for 1 h, and then incubated with anti-TET1(MA5-16312, 1:1000 dilution, Thermo Fisher Scientific) or anti-β-actin (ARG65683, 1:1000 dilution, Arigo) antibodies for 2 h, followed by incubation with horseradish peroxidase-conjugated goat anti-rabbit IgG for 1 h. Protein bands were visualized by an ECL + Plus western blotting detection system (CW Biotech, Beijing, China) and quantified using a scanner with Quantity One software (Version 4.2.1, Bio-Rad Laboratories, Hercules, CA, USA).

### Total RNA extraction and real-time PCR (qRT‐PCR)

Total RNA was extracted from PCa cell lines and clinical tissues using TRIzol reagent (Invitrogen, Carlsbad, CA, USA) and reverse transcribed into cDNA using the RevertAid™ first strand cDNA synthesis kit (Thermo Fisher Scientific, USA). The obtained cDNA was subjected to qRT-PCR by performing on a LightCycler480®II in a final volume of 25 µL. Optimum reaction conditions were obtained with 0.04 µL of 100 µM of each primer and probe, 2.5 µL of 10× PCR buffer, 2.5 µL of 25 mM MgCl_2_, 0.5 µL of 10 mM 4× dNTPs, 0.25 µL of 5 U/µL Taq DNA polymerase, and 2 µL template cDNA. Finally, 17.13 µL ddH_2_O was added to the reaction mixture. The mixture was preheated at 95ºC for 3 min to activate Taq polymerase, followed by 40 cycles at 95ºC for 5 s and 60ºC for 15 s. Samples were amplified simultaneously in triplicate in one assay run. Data were normalized to GAPDH or U6. Sequences of primers and probes are summarized in Table 1.

**Table 1:** Sequences of primers and probes for real-time RT-PCR

Gene	Primer / Probe	Sequence (5’ to 3’)
miR-183-5p	Forward	CCTGTTCTGTGTATGGCACTGGT
	Reverse	TTCACTGACTGAGACTGTTCACAGTG
Human TET1	Forward	CCATCTGTTGTTGTGCCTCTG
	Reverse	GCCATTTACTGGTTTGTTGTCA
	Probe	FAM-AGGTTATAAAGGAAAACAAGAGGCCCC-BHQ-1
Human GAPDH	Forward	CAACTACATGGTTTACATGTTC
	Reverse	GCCAGTGGACTCCACGAC
	Probe	CY5-TTTGGTCGTATTGGGCGCCTG-BHQ-1

### Statistically analysis

Data were presented as mean ± S.E.M., and GraphPad Prism (version 5.0) was used for data analyses.

The paired Student’s t-tests were performed to compare two related samples, while the unpaired Student’s t-tests were used to compare differences between different groups. A one-way ANOVA test was conducted to compare the intergroup difference more than two groups. Kaplan– Meier curves were introduced for survival analysis. *P* value less than 0.05 was considered as statistically significant.

## Results

### Up-regulated expression of mir-183-5p in PCa tissues

**Fig. 1:**
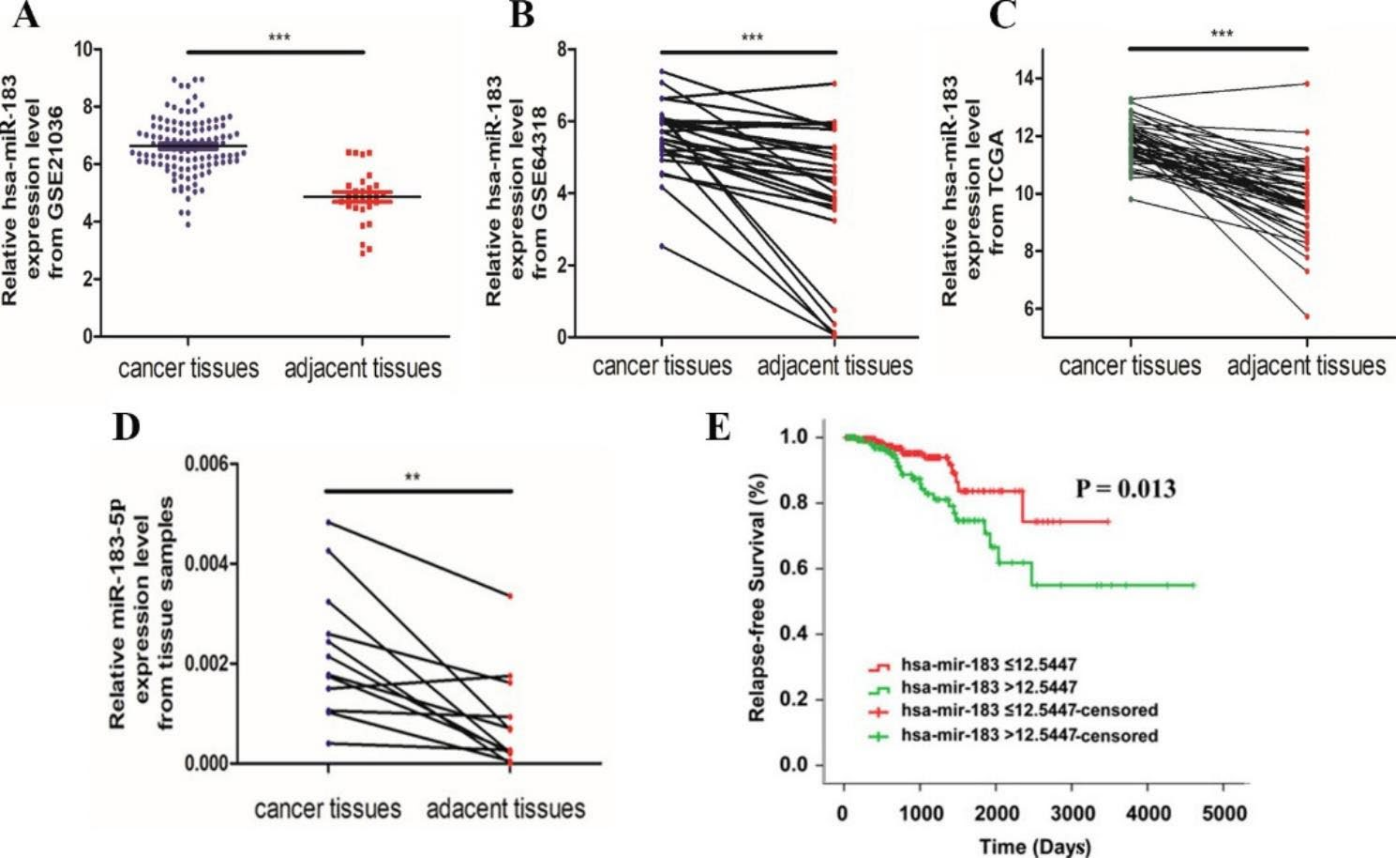
Relative miR-183 expression was up-regulated in human PCa tissues. **(A)** MiR-183 mRNA level in PCa samples from GEO database (GSE21036). **(B, C)** MiR-183 expression level in PCa samples and paired adjacent tissues from GEO database (GSE64318) and TCGA database. **(D)** Relative expression level of miR-183-5p in human PCa tissues was compared with that in adjacent tissues (12 cases). **(E)** Relapse-free survival curve of PCa patients based on miR-183 from TCGA datebase (n = 495). Data are presented as mean ± SEM; ^***^P < 0.001; ^**^P < 0.01; ^*^P < 0.05

To investigate the expression level of miR-183-5p in PCa, we collected miRNA expression profiles of PCa patients from the TCGA and GEO database. We found that the expression of miR-183 was significantly increased in PCa tissues (Fig. 1). Data from microarray datasets (GSE21036 and GSE64318) showed miR-183 expression was remarkably upregulated in PCa tissues compared to that in adjacent tissues (Fig. 1A, B), these differences were consistent with the results obtained from TCGA(Fig. 1C). To further validate the miR-183-5p expression level in PCa tissues, we examined the expression in 12 pairs clinical specimens of prostate cancer by qRT-PCR. The results showed that miR-183-5p was highly expressed in PCa tissues, which was consistent with the above (Fig. 1D). In addition, Kaplan-Meier analysis showed that PCa patients with high miR-183 expression had shorter recurrence-free survival than those with low miR-183 expression (Fig. 1E). Therefore, all the above indicate that miR-183-5p may serve an important role in the development and progression of PCa.

### MiR-183-5p mimic transfection promotes migration and invasion of PCa cell lines

**Fig. 2:**
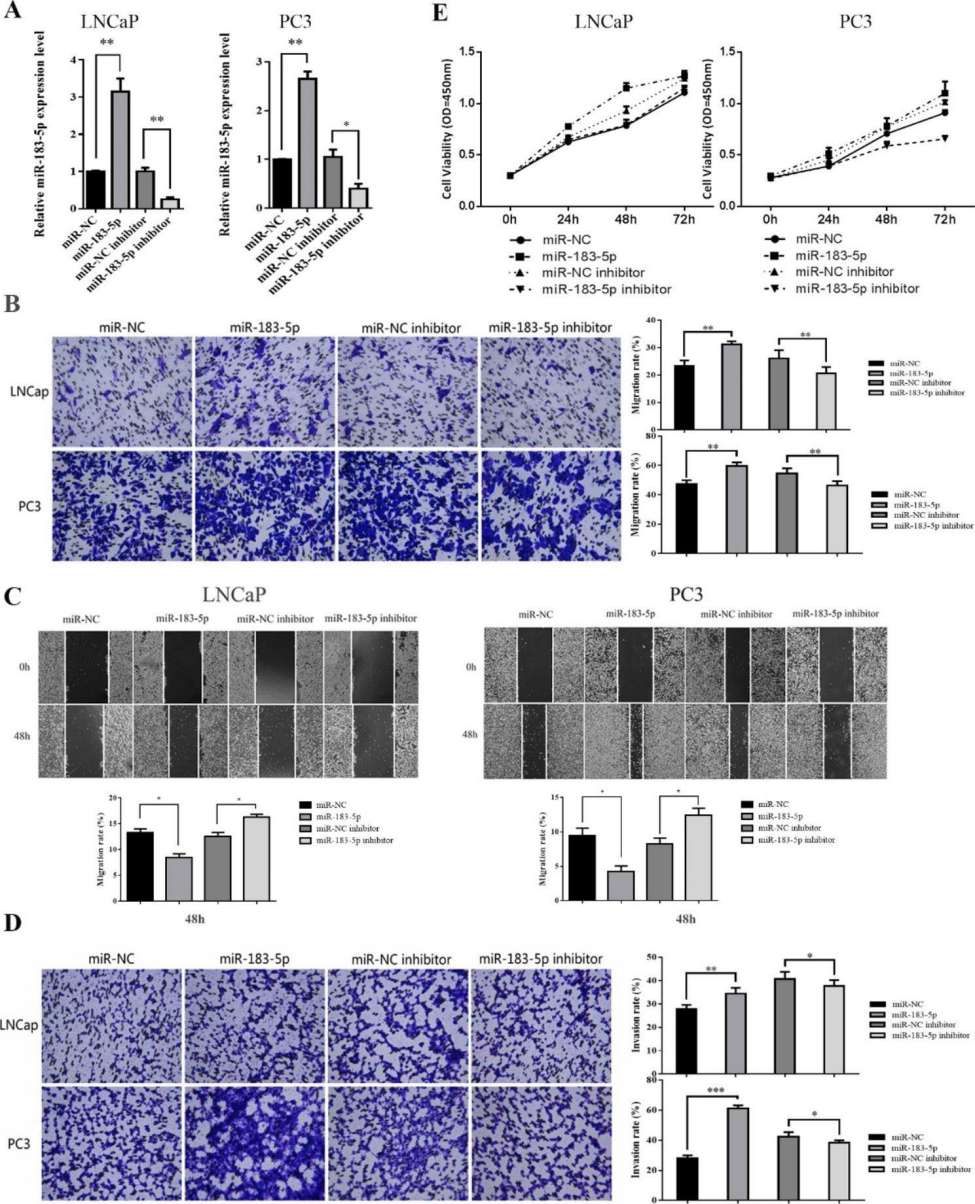
MiR-183-5p promoted the migration and invasion of PCa cell lines. **(A)** qRT-PCR was used to verify the transfection efficiency of miR-183-5p after transfection of miR-NC, miR-183-5p, miR-NC inhibitor and miR-183-5p inhibitor in PC3 and LNCaP cell lines. **(B)** The migration(vertical) of the two transfected PCa cell lines was measured using a transwell assay (magnification: 10×). **(C)** The crawling ability of the transfected PCa cell lines was measured using a wound healing assay (magnification: 10×). **(D)** The invasion of the transfected PCa cell lines was measured with transwell assay (magnification: 10×). **(E)** Cell proliferation of the transfected PCa cell lines was measured with CCK-8 assay. Data are presented as mean ± SEM; ^***^P < 0.001; ^**^P < 0.01; ^*^P < 0.05

To study the biological function of miR-183-5p in PCa cell lines, overexpression or inhibition of miR-183-5p was transfected into two prostate cancer lines LNCaP and PC-3, respectively (Fig. 2A). Results obtained showed the horizontal and vertical migration abilities of both LNCaP and PC3 cells were notably enhanced following miR-183-5p mimic transfection, while alleviated by miR-183-5p inhibitor transfection (Fig. 2B, C). Besides, transwell assay revealed similar results (Fig. 2D). However, there were no significant differences on the cell proliferation among different groups of both LNCaP and PC3 cells (Fig. 2E). All these results indicated that miR183-5p could promote the migration and invasion of PCa cell lines.

### MiR-183-5p targets TET1 in prostate cancer

**Fig. 3:**
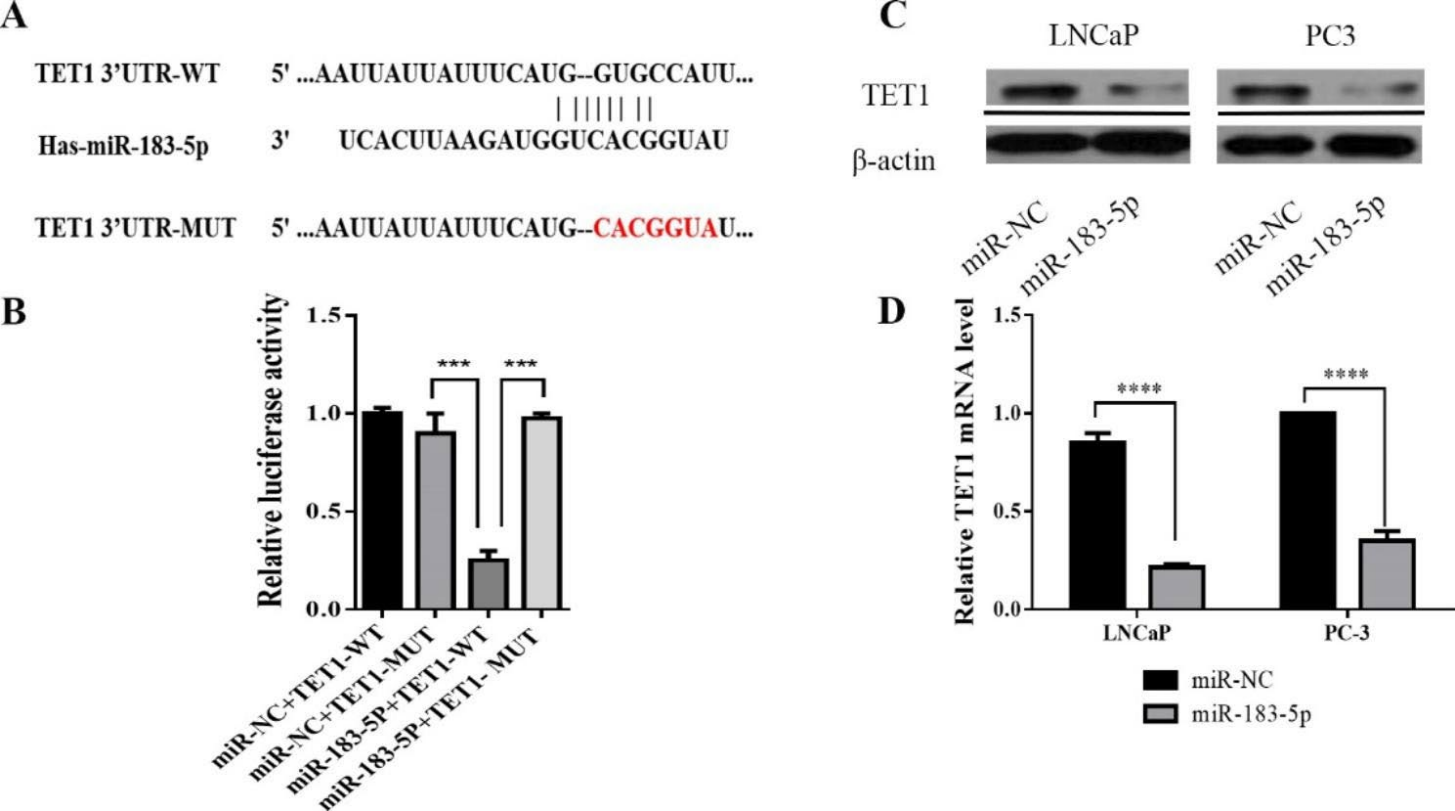
MiR-183-5p could bind to 3’-UTR of TET1. **(A)** Binding sites of miR-183-5p in the TET1 3’-UTR predicted by TargetScan. WT: wild type; MUT: mutated type. The mutant nucleotides are in red. **(B)** The direct targeting of miR-183-5p to TET1 was confirmed by dual luciferase reporter assays. **(C)** TET1 protein levels were analyzed using western blot in PCa and LNCaP cell lines after transfection of miR-NC and miR-183-5p mimic, respectively, and β-actin was a loading control. Western blots were cropped for clarity, uncropped images are shown in Supplementary material. **(D)** TET1 mRNA levels was determined by qRT-PCR in PC-3 and LNCaP cells after transfection of miR-NC and miR-183-5p mimic, respectively. Data are presented as mean ± SEM; ^****^P < 0.0001; ^***^P < 0.001

MiRNAs exert their biological functions primarily by degrading or inhibiting their target mRNAs. In order to explore the potential target gene of miR-183-5p, software including TargetScan, miRDB, miRWalk database was used to predict proper molecules. The website indicated the presence of a binding site between the sequence of miR-183-5p the 3’-UTR of TET1(Fig. 3A), subsequently luciferase reporter assay was performed to confirm whether TET1 was a direct target of miR-183-5p. The results showed the luciferase activity of TET1 WT 3’-UTR but not TET1 MUT 3’-UTR was remarkably reduced following miR-183-5p mimic transfection (Fig. 3B), suggesting that miR-183-5p targets TET1.

Moreover, western blotting and qRT-PCR were performed to confirm the targeted relationship between miR-183-5p and TET1. The results showed the expression levels of TET1, both mRNA and protein, were significantly decreased in PC-3 and LNCaP cells transfected with miR-183-5p mimics, compared to mimic NC (Fig. 3C, D). Taken together, these data indicated that miR183-5p negatively regulated TET1 expression in PCa cell lines.

### Upregulation of TET1 partially reversed the effects of miR-183-5p in PCa cell lines

**Fig. 4:**
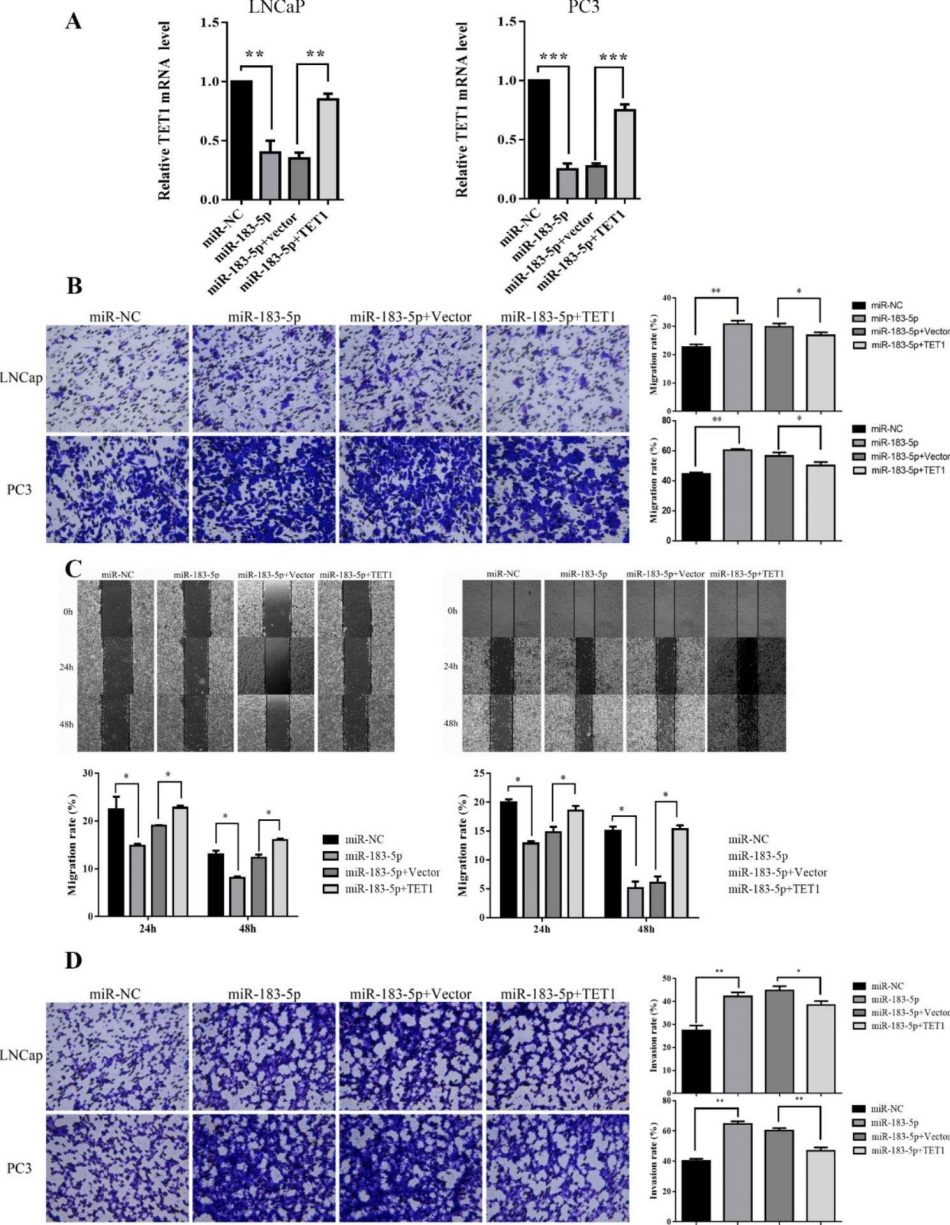
MiR-183-5p facilitated the migration and invasion of PCa cells through down-regulation TET1. **(A)** The expression level of TET1 in the co-transfected PCa cell lines of miR-183-5p and TET1 was detected by qRT-PCR. **(B)** The migration(vertical) in the two co-transfected PCa cell lines of miR-183-5p and TET1 was measured using a transwell assay (magnification: 10×). **(C)** The crawling ability in the two co-transfected PCa cell lines of miR-183-5p and TET1 was measured using a wound healing assay (magnification: 10×). **(D)** The invasion in the two co-transfected PCa cell lines of miR-183-5p and TET1 was measured with transwell assay (magnification: 10×). Data are presented as mean ± SEM; ^***^P < 0.001; ^**^P < 0.01; ^*^P < 0.05

To further explore whether the acceleration of PCa cell lines migration and invasion mediated by miR-183-5p was regulated by TET1, TET1 overexpression plasmid was constructed and co-transfected into both LNCaP and PC3 cells with miR-183-5p mimic or miR-NC. qRT-PCR assay demonstrated that the mRNA expression level of TET1 was upregulation after co-transfection of TET1 and miR-183-5p mimic compared with the control group transfected with miR-183-5p mimic alone (Fig. 4A). Additionally, overexpression of TET1 was able to counteract the accelerated migration and invasion of miR-183-5p mimic on PCa cells by wound healing assay and transwell assay (Fig. 4B, C, D).

## Discussion

The present study aimed to investigate the regulatory role of miR-183-5p and its potential target TET1 in the progression of PCa. In clinical studies, we found that miR-183-5p was up-regulated in PCa tissues, moreover, PCa patients with high miR-183 expression had shorter recurrence-free survival than those with low miR-183 expression. While in functional studies, we observed that overexpression of miR-183-5p promoted the migration and invasion of PCa cells, both LNCaP and PC3 cells. Meanwhile, miR-183-5p down-regulated the expression of TET1 protein in cells. On the contrary, inhibit miR-183-5p retarded migration and invasion processes of PCa cells and increase the expression of TET1 protein. By constructing a luciferase reporter vector, we determined that TET1 was a direct target of miR-183-5p.

MiR-183-5p is located on chromosome 7q31-34, which is a member of the miR-183-96-182 cluster. This cluster includes miR-183, miR-96 and miR-182, the three members have highly homologous sequence. The miR-183-96-182 cluster was originally identified as a sensory organ-specific miRNA cluster, including hearing, vision and olfaction [28, 29, 31, 32]. However, recent studies have demonstrated that the miR-183-96-182 cluster was involved in oncogenesis and cancer progression[33]. Among them, miR-183-5p was reported highly expressed in renal cell carcinoma, non-small cell lung cancer and breast cancer, and promoted malignant progress, even resulting in radioresistance [34–37]. Consistent with above, miR-183-5p was found overexpression in PCa tissues and it positively regulated serum levels of PSA, which functioned as an oncogene in PCa [15, 38]. This conclusion was further supported by our findings in this study. Moreover, our findings show for the first time that miR-183-5p can also directly modulate TET1 expression in PCa, which suggesting that miR-183-5p may affect tumorigenesis and progression through DNA methylation.

TET1 has been recognized as a tumor suppressor in a variety of human cancer[39, 40]. It had been verified that the protein and mRNA levels of TET1 were decreased in PCa, and low TET1 mRNA levels correlated significantly with a wore metastasis-free survival[41]. In addition, in xenograft models, TET1 deficiency facilitated tumor growth and metastasis[42]. These results implied that TET1 might participate in the pathogenesis of PCa. Carolina et al. showed that miR-183-5p could strengthen the ability of PCa cells to adhesion via repression of ITGB1 expression[21]. Here we found that overexpression of miR-183-5p enhanced the malignant phenotype of PCa cells by inhibiting TET1 expression. Our studies have further confirmed that miRNAs and their target genes did not have a one-to-one correspondence, at times, one miRNA would target different mRNAs, and one target mRNA would be regulated by serval miRNAs in the same human tumor.

But there were still some findings not in accord with the expectation. We found up-regulation of miR-183-5p promoted the migration and invasion of PCa cells, but did not affect cell proliferation. However, Larne et al. found that overexpression of miR-183 induced cell growth in PCa cells[15]. We suspect that this may be due to distinct biological effects of miR-183-5p and miR-183-3p.Two mature miRNAs, deriving from 3’ and 5’ ends of the same pre-miRNA, may induce different effect in proliferation, migration and invasion[43]. Ling et al. reported that phoenixin-14 regulated proliferation and apoptosis of vascular smooth muscle cells by activating KCNQ1OT1/miR-183-3p/CTNNB1 pathway, which partly confirmed our speculation[44]. Nevertheless, it is true that more research is needed to definite the biological functions of the miR-183 hairpin RNA products, respectively, in order to obtain a better understanding of the regulatory mechanism of miR-183 in PCa.

Briefly, miR-183-5p promotes the migration and invasion of PCa cells, and high expression of miR-183 is associated with poor prognosis of PCa patients. In addition, miR-183-5p targeting TET1 may be a new potential biomarker for PCa.

## Electronic supplementary material

Below is the link to the electronic supplementary material.

Additional File: Uncropped western blot images for Fig 3C

## Data Availability

Gene expression and clinical data of patients with prostate cancer in PRAD-TCGA were downloaded from UCSC Xena Cancer browser (https://xenabrowser.net). Microarray datasets (GSE21036 and GSE64318) were downloaded from Gene Expression Omnibus (GEO) (https://www.ncbi.nlm.nih.gov/gds/). There are no restrictions on data availability.
