# Supplementary material for: MiR-183-5p promotes migration and invasion of prostate cancer by targeting TET1
Source: BMC Urol. 2023 Jul 10;23:116. doi: 10.1186/s12894-023-01286-7 (PMC10334645; doi:10.1186/s12894-023-01286-7)
Supplement: Supplementary file 1 — Additional File: Uncropped western blot images for Fig 3C [file 12894_2023_1286_MOESM1_ESM.pdf]

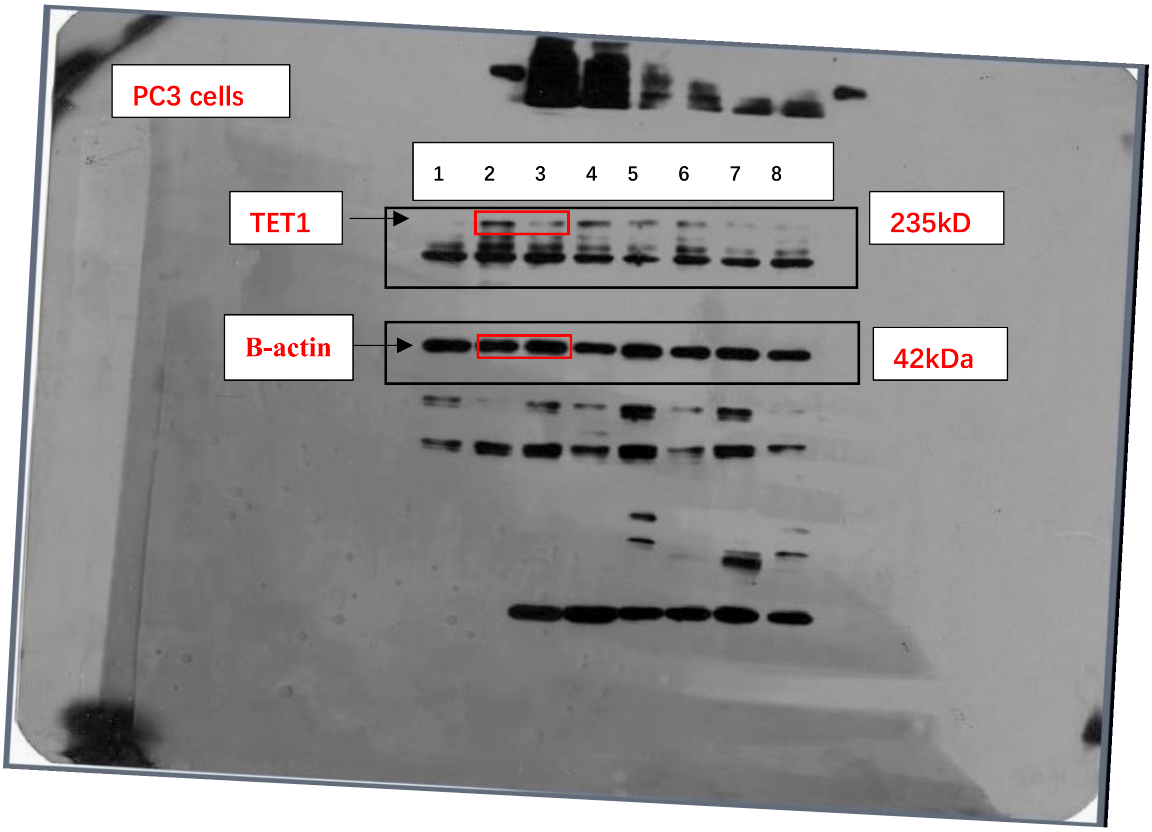

MiR-NC: Lane 2, 4, 6

MiR-183-5p: Lane 1, 3, 5, 7, 8

L

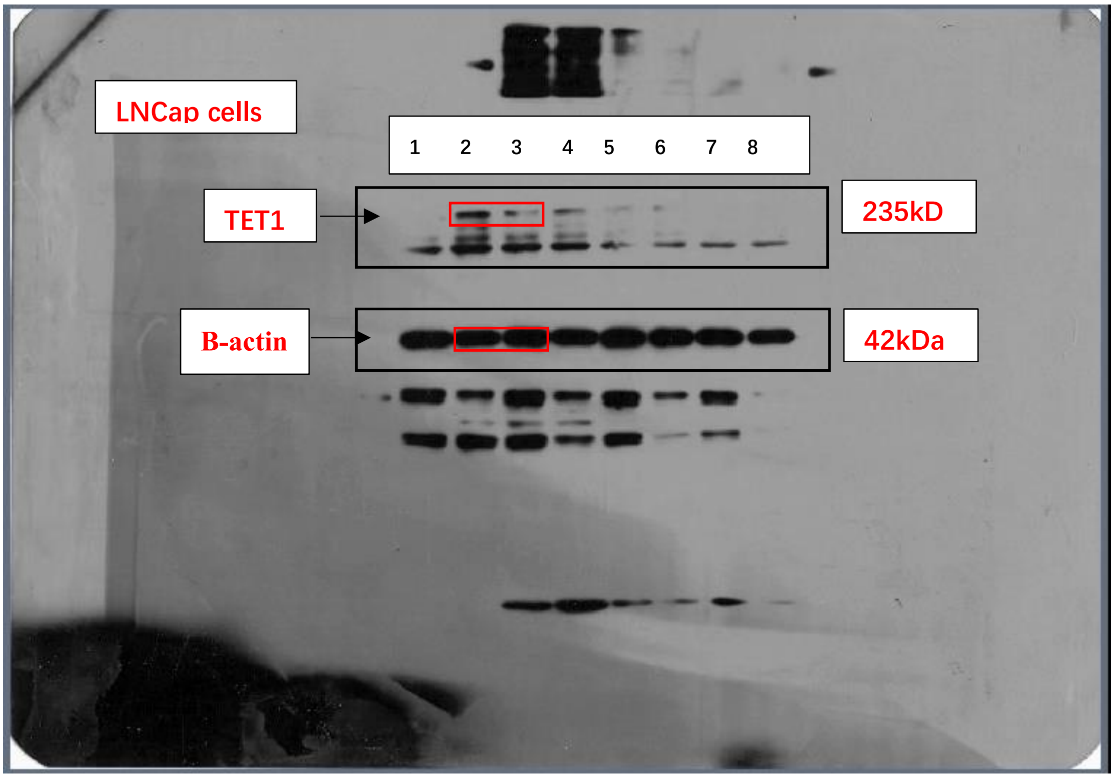

MiR-NC: Lane 2, 4, 6

MiR-183-5p: Lane 1, 3, 5, 7, 8

The protein bands used in the article were exposed with X-ray film, and a gray background was used as requested. Due to the bands around 250 kDa (the position of TET1) were shallow, we extended exposure time until the weakest bands appeared. The borders of the blot have been indicated by a black line, and the bands marked in the red box showed in Figure 3C.
